# Production of *Vespa tropica* Hyaluronidase by *Pichia pastoris*

**DOI:** 10.3390/jof10120854

**Published:** 2024-12-11

**Authors:** Piyapon Janpan, Bernhard Schmelzer, Anuwatchakij Klamrak, Patthana Tastub, Tewa Upathanpreecha, Shaikh Shahinur Rahman, Jaran Nabnueangsap, Yutthakan Saengkun, Prapenpuksiri Rungsa, Diethard Mattanovich, Sakda Daduang

**Affiliations:** 1 Division of Pharmacognosy and Toxicology, Faculty of Pharmaceutical Sciences, Khon Kaen University, Khon Kaen 40002, Thailand; j.piyapon@kkumail.com (P.J.); anuwat_kla@yahoo.com (A.K.); shahinanft@gmail.com (S.S.R.); yutthakan_s@kkumail.com (Y.S.); 2 Protein and Proteomics Research Center for Commercial and Industrial Purposes (ProCCI), Khon Kaen University, Khon Kaen 40000, Thailand; prapen_rungsa@kkumail.com; 3 Institute of Microbiology and Microbial Biotechnology, Department of Biotechnology, University of Natural Resources and Life Sciences, Vienna (BOKU), 1190 Vienna, Austria; bernhard.schmelzer@gmail.com (B.S.); diethard.mattanovich@boku.ac.at (D.M.); 4 Betagro Science Center Co., Ltd., 136 Khlong Nueng, Khlong Luang District, Pathum Thani 12120, Thailand; patthanat@betagro.com (P.T.); tewa_u@betagro.com (T.U.); 5 Department of Applied Nutrition and Food Technology, Faculty of Biological Sciences, Islamic University, Kushtia 7000, Bangladesh; 6 Salaya Central Instrument Facility RSPG, Research Management and Development Division, Office of the President, Mahidol University, Nakhon Pathom 73170, Thailand; jaran.nab@mahidol.edu

**Keywords:** hyaluronidase enzyme, *Vespa tropica*, *Pichia pastoris*

## Abstract

Hyaluronidases have been a subject of great interest in medical and cosmeceutical applications. Previously, our group demonstrated that the venom glands of *Vespa tropica* contain hyaluronidase enzymes (VesT2s), and heterologous expression of the corresponding gene (*VesT2a*) in *E. coli* systems results in inclusion bodies, necessitating functional folding using urea. Here, we report the successful heterologous expression of VesT2a in the *Pichia pastoris* expression system, with gene construction achieved using Golden*PiCS*. After confirming gene integration in the yeast genome, methanol-induced cultures yielded an exceptional amount of VesT2a, approximately two-fold higher than that obtained with the constitutive expression vector (P_GAP_). Upon culturing in a bioreactor, yeast cells harboring pAOX1-αMF-*VesT2a* produced secreted proteins with a total yield of 96.45 mg/L. The secreted VesT2a has a molecular weight of 59.35 kDa, significantly higher than the expected molecular weight (~40.05 kDa), presumably due to endogenous glycosylation by the yeast cells. It exhibits optimal activity at 37 °C and pH 3, showing a specific activity of 4238.37 U/mg, and remains active across a broad range of pH and temperature. Notably, it exhibits higher hyaluronidase activity than the crude venom and *E. coli*-expressed protein, likely due to improved folding via endogenous post-translational modifications, such as disulfide bonds and N-glycosylation; this underscores the potential of heterologous systems for producing venomous hyaluronidases from other species. In silico docking-based analyses further support its catalytic activity and provide insights into seeking natural inhibitors from phenolic-rich plant extracts to alleviate symptoms in patients suffering from insect bites and stings.

## 1. Introduction

Hyaluronidases (Hyals) are glycosidase enzymes that catalyze the specific cleavage of β-N-acetyl-D-glucosaminidic bond in the hyaluronic acid polymer to give various chain lengths of hyaluronan oligosaccharides such as di-, tetra-, and hexamers [1,2]. According to the catalytic activities, with certain types of degraded products generated, they can be classified to certain groups, including hyaluronate 4-glucanohydrolase (EC 3.2.1.35), leech hyaluronidase (EC 3.2.1.36), and microbial hyaluronidases (EC 4.2.2.1) [3]. Living organisms produce these enzymes for ecological and biological reasons. Microorganisms (e.g., *Streptococcus pyogenes*, *Candida albicans,* and *Streptomyces coelicolor*) secrete this enzyme to utilize the environmental hyaluronan, where the degraded products, in particular N-acetyl glucosamine (GlcNAc), serve as a carbon source [4,5]. Concerning the immunological roles, human white blood cells (WBCs) secrete hyaluronidase to facilitate their passage through connective tissue to reach the infected locations [6]. Sperm hyaluronidase has been implicated to play a significant role in fertilization processes in mammals [7]. 

Like other organisms, many venomous animals produce various forms of Hyals for hunting, self-defense, and protecting others [8]. These enzymes have also been applied in medicine as permeation enhancers, improving the bioavailability of numerous anticancer and tumor-targeting drugs [9,10,11]. The daratumumab and hyaluronidase-fihj combination injection (Darzalex Faspro™) is used alongside bortezomib, melphalan, and prednisone to treat newly diagnosed multiple myeloma in patients ineligible for autologous stem cell transplantation [12]. Similarly, trastuzumab and hyaluronidase-oysk (Herceptin Hylecta™) are paired with various cancer treatments to target HER2-overexpressing breast cancer [13]. Hyals from the venom glands of insects (e.g., *Buthus martensi* and *Vespula* species) show anticancer and immunotherapeutic properties [14,15], while bovine testicular hyaluronidase (Hylase Dessau^®^) is widely applied in aesthetic dermatology [16]. Notably, Hyals’ degraded products (e.g., decamers and tetramers) have also been found to simultaneously disrupt HA-CD44 interactions, exhibiting cytotoxicity against breast cancer and showing promise for combating bone metastasis [17]. These applications underscore the need for microbial cell factories to produce Hyals as permeation enhancers while generating HA oligomers for anticancer purposes.

*Vespa tropica *(greater banded hornet), one of the most dangerous insects in the world, uses its potent sting in hunting due to the presence of various chemicals and spreading virulent factors such as phospholipase (33.33%), hyaluronidase (25.13%), antigen 5 (10.33%), and dipeptidyl peptidase (9.00%), respectively. The crude venom of this venomous insect exhibits remarkably high specific hyaluronidase activity over other poisonous animals including *Vespa affinis*, *Heterometrus laoticus,* and *Naja siamensis,* and is significantly comparable to the bovine testicular hyaluronidase [18]. Our group succeeded in achieving the isolation of the gene encoded for hyaluronidase (VesT2a) from the venom gland of *V. tropica*, whose open reading frame (ORF) consisted of 1486 bp (356 amino acids) with a theoretical mass of 39,119.73 Da/pI 8.91 [19]. Multiple sequence amino acids alignment revealed two crucial catalytic residues (Asp107 and Glu109), five putative glycosylation regions (Asn79, Asn99, Asn127, Asn187, and Asn325), and two disulfide bridges (C19-C308 and C185-C197) appear in the VesT2a amino acid sequence. Unfortunately, the recombinant VesT2a proteins overproduced by *E. coli* expression systems were detected as inclusion bodies, which required chemical refolding using urea, and were found to have the lower specific hyaluronidase activity (approx. 3-folds) as compared to its relevant crude venom. This result is consistent with the heterologous expression of hyaluronidase from *P. paulistra* and *V. affinis* using *E. coli* systems, which leads to inclusion body formation as well [20,21]. We hence speculate that the chemical-assisted refolding is not sufficient to allow a proper folding and function of our target protein and may require post-translational modifications (e.g., glycosylation and disulfide bridge formation) through the use of eukaryotic expression systems.

Engineering yeast, as a microbial chassis to produce recombinant protein, provides many benefits over bacterial systems [22,23,24,25,26,27]. Among methylotrophic yeasts, we especially highlight the following: (1) *Pichia pastoris*, also known as*Komagataella phaffii,* is engineerable to secrete the target agents (e.g., peptides and proteins) into surrounding medium by fusing with the proper leader sequences derived from α-mating factor and human serum albumin (HSA), reducing breaking cells and purification steps when compared to those of mammalian hosts (e.g., CHO and HEK 293). (2) *P. pastoris* exhibits a high growth rate and it does not require an expensive medium, enabling large-scaled production. (3) *P. pastoris* is permitted by the FDA as it is generally recognized as safe (GRAS) because it does not contain any endotoxins, harmful substances, or viruses. (4) Heterologous expression of recombinant proteins could be conducted simply by supplying methanol as an inducer into the culture, turning on gene expression regulated tightly by alcohol oxidase (*AOX1*) promoter. (5) Other constitutive expression vectors (e.g., pGK1 and pGAPZ) have also been used for driving the expression of target genes in *P. pastoris*, without relying on the external inducers. (6) Besides its own homologous recombination that is basically used, an efficient integration of the target DNA into the yeast genome could be enhanced by using recombinase-based gene integration approach [28,29]. More importantly, methylotrophic yeast can proceed in post-translation modifications, such as glycosylation and disulfide bond formations, which are vital for the structural integrity and biocatalytic activity of many proteins [30,31]. Previous studies have demonstrated their achievements in producing bee, scorpion, and leech hyaluronidases using *P. pastoris*, in which they were secreted into the culture mediums and exhibited strong hyaluronidase activity over those present in their crude venoms [32,33,34].

**Figure 1:**
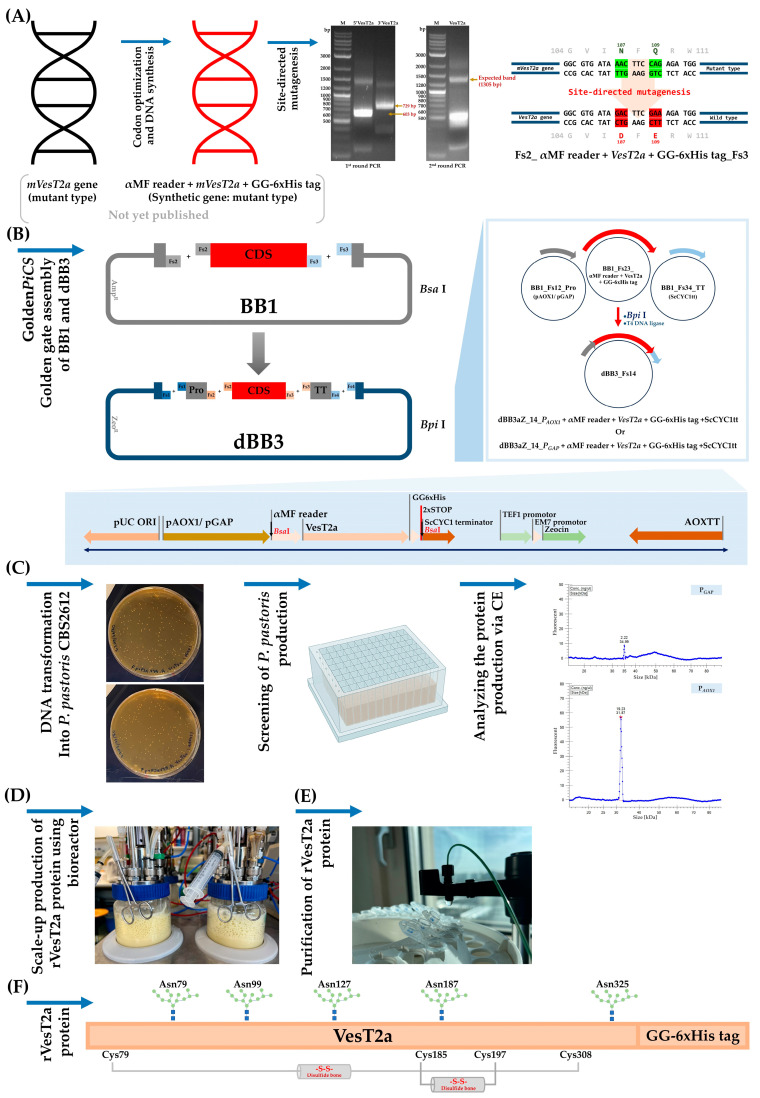
Schematic illustration of the protein production steps for VesT2a from *V. tropica* using the *P. pastoris* system: (**A**) codon optimization and site-directed mutagenesis techniques were used to prepared the wild-type *VesT2a* gene; (**B**) recombinant plasmid construction via the Golden*PiCS* system; (**C**) recombinant *VesT2a* plasmid integration into *P. pastoris* and screening of *P. pastoris* production using enzymatic glucose release method before analyzing by capillary gel electrophoresis; (**D**) upscaling VesT2a protein production using a bioreactor; (**E**) protein purification through affinity chromatography; (**F**) VesT2a protein structure depiction.

Here, *P. pastoris* was used as a cell factory to produce the codon-optimized version of the *VesT2a* gene. Gene construction was achieved using Golden*PiCS*, a Golden-Gate-derived modular cloning system specifically designed for heterologous gene expression in the *P. pastoris* expression system (Figure 1). The effect of different promoters on the expression of VesT2a was investigated. In addition to the SDS-PAGE and Western blot analyses, the secreted VesT2a protein was characterized by the LC-MS/MS technique. The yeast-derived VesT2a exhibited strong hyaluronidase activity over those obtained from *the E. coli* system and the crude venom of *V. tropica*. Two phytochemicals, quercetin and gallic acid, were used as hyaluronidase inhibitors in a hyaluronidase activity assay to be utilized as an alternative method to analyze the secreted protein. Molecular docking was employed to explore the interaction between VesT2a and its ligands, e.g., hyaluronic acid and inhibitors, shedding light on protein engineering for the foreseeable future. Strategies reported herein could be applied for the production of insect-derived Hyals and other bioactive enzymes and constituents originating from poisonous organisms to serve medical and cosmeceutical purposes.

## 2. Materials and Methods

### 2.1. Strains, Reagents, and Medias 

All microbial strains (e.g., *E. coli* DH10B, *P. pastoris* CBS 2612), reagents, and backbone DNA for Golden*PiCS* system were kindly provided by Diethard Mattanovich (BOKU, Vienna, Austria). The synthetic gene (gBlock), which is FS2_α-MF_*mVesT2a*_GG-6xHis tag_FS3, was purchased from TWIST bioscience (South San Francisco, CA, USA). The specific primers that are shown in Table 1 were purchased from Integrated DNA Technologies IDT (Coralville, IA, USA).

### 2.2. Site-Directed Mutagenesis

The hyaluronidase (*VesT2a*) gene was performed via an overlapping extension PCR of the site-directed mutagenesis technique. In the first round of PCR, a synthetic gene (Fs2_α-MF_*mVesT2a*_GG-6xHis tag_FS3) served as the DNA template, with two specific primer sets, F_VesT2a with R_SDM, and F_SDM with R_VesT2a (Table 1), to amplify two initial DNA fragments, using Q5 High-Fidelity DNA Polymerase (NEB, Ipswich, MA, USA). The thermocycling condition included an initial denaturation at 98 °C for 30 s, followed by 30 cycles of 98 °C for 10 s, 70 °C for 30 s, and 72 °C for 45 s, with a final extension at 72 °C for 2 min. Both PCR products were purified using the innuPREP DOUBLEpure Kit (Analytik Jena, Jena, Germany). In the second PCR round, the purified PCR products were used as DNA templates, along with a specific primer, F-VesT2a and R-VesT2a, to generate the CDS fragment (Fs2_α-MF_*VesT2a*_GG-6xHis tag_Fs3) using Q5 High-Fidelity DNA Polymerase. 

### 2.3. Gene Cloning via the Golden-Gate-Derived P. pastoris Cloning System (GoldenPiCS) 

The Golden*PiCS* system was utilized to construct recombinant plasmids for the hyaluronidase gene expression in *P. pastoris* (Table 2), following [35].

Golden Gate assembly—BB1: The CDS fragment was inserted into recipient empty backbone 1 (eBB1_Fs-23) using *Bsa*I enzyme, and T4 DNA ligase. The GGA reaction was incubated with 30 cycles of 37 °C for 1 min and 16 °C for 1 min, and a final incubation at 37 °C for 10 min. The BB1 plasmid (BB1_Fs23_α-MF_*VesT2a*_GG-6xHis tag) was then transformed into *E. coli* DH10B using heat shock method at 42 °C for 70 s. Transformants containing the target gene were selected on LB agar with 25 µg/mL kanamycin. The BB1 plasmid was then extracted by HiYield^®^ Plasmid Mini Kit (Süd-Laborbedarf GmbH, Gauting, Germany), and verified by Sanger Cycle Sequencing with F_BB1 and R_BB1 primers (Microsynth AG, Vienna, Austria). 

Golden Gate assembly—dBB3: Single transcription unit inserted in BB1 plasmid, promoters (BB1_12_pGAP or BB1_12_pAOX1), and terminator (BB1_34_ScCYC1tt) were assembled into the recipient empty direct backbone 3 (edBB3aZ_Fs-14) using *Bpi*I enzyme and T4 DNA ligase to construct two BB3 plasmids, BB3aZ_Fs-14_pAOX1_αMF_*VesT2a*_GG-6xHis tag_ScCYC1tt and BB3aZ_Fs-14_pGAP_αMF_*VesT2a*_GG-6xHis tag_ScCYC1tt, before transforming into *E. coli* DH10B. Transformants were selected on LB agar containing 50 µg/mL zeocin and verified the desired gene by Sanger Cycle Sequencing with F_AOX1 and R_BB3 primers or F_GAP and R_BB3 primers. The schematic maps of the recombinant hyaluronidase gene plasmids (dBB3) were generated using GenSmart Design (accessed on 27 August 2024 at https://www.genscript.com/gensmart-design/#) (Figure S1). Subsequently, the selected clones of each BB3 plasmid were linearized with the *Asc*I enzyme and integrated into the *P. pastoris* chromosome via the electroporation method. The transformation reactions were plated on YPD agar containing 500 µg/mL zeocin and incubated at 28 °C for 48 h. 

### 2.4. Screening of P. pastoris Production Using Enzymatic Glucose Release Method

The screening of recombinant hyaluronidase protein production by *P. pastoris* was performed using enzymatic glucose release method in 96 deep-well plates (96-DWPs). The Enpump200 kit (Enpresso, Berlin, Germany) was used to apply in the slowing glucose release condition. The randomly yeast transformants of each plasmid construct were inoculated in 300 µL YPD containing 500 µg/mL zeocin at 25 °C, 1200 rpm for overnight. Each inoculated plate culture was then centrifuged at 2000× *g* for 5 min to separate pellet cells and supernatant. The pellet cells were resuspended with 150 µL of 2xASM.V6 minimal media (6.3 g/L (NH_4_)_2_HPO_4_, 0.8 g/L (NH_4_)_2_SO_4_, 0.49 g/L MgSO_4_·7H_2_O, 2.64 g/L KCl, CaCl_2_·2H_2_O, 22 g/L citric acid monohydrate, 1.47 mL/L PTM1 trace metals, and 20 mL/L NH_4_OH (25%); pH set to 6.5 with KOH). Subsequently, 30 µL of suspended culture was transferred into a new set of 96-DWPs with 120 µL of 2xASM.V6 minimal media. 

For P_GAP_ screening, 150 µL of PSE solution containing 50 g/L EnPump200 substrate (polysaccharide solution; PS) and 0.7% amylase was added into the main culture to achieve glucose limiting conditions. Then, the cultures were incubated at 25 °C, 1200 rpm for 48 h. 

For P*_AOX1_* screening, 150 µL of PSE solution, containing 25 g/L EnPump200 substrate (polysaccharide solution; PS) and 0.35% amylase, was added to each well of a 96-DWP with the main culture and incubated at 25 °C and 1200 rpm. At 3, 19, 27, and 43 h, 10, 20, 20, and 20 µL of absolute methanol, respectively, were added to each well to induce recombinant protein expression.

After incubating about 48 h, the protein production screening of VesT2a protein by *P. pastoris* from different constructions were analyzed using a gel capillary electrophoresis technique with LabChip^®^ HT Protein Express Chip (PerkinElmer, Waltham, MA, USA). The candidate clone, which has the potential to produce VesT2a protein, was chosen for upscaled production using a bioreactor system.

### 2.5. Bioreactor Cultivation

Bioreactor cultivation with a sequential induction strategy was carried out using the DASGIP^®^ Parallel Bioreactor System (Eppendorf, Hamburg, Germany) [36]. The process began with the batch phase, where 300 mL of modified BSM media containing 4% glycerol as a carbon source and 10 mL of inoculated culture was added to the reactor. The cultivation was controlled at 25 °C, with dissolved oxygen (DO) at 20% regulated by stirrer speed, pH 5.5 adjusted by 25% NH_4_OH, and 5% glanapon used to prevent foam formation. After approximately 18 h, signaling the end of the batch phase, phase two started with the addition of a glucose-feeding medium containing 50% glucose, 1% biotin, and 1% PTM0; this was pumped into the reactor for 24 h at an exponential flow rate defined by the equation y = 1.3465e^0.0606x^ for a specific growth rate (µ) of 0.061 h^−1^. Following glucose depletion, the heterologous protein production was initiated by methanol adding, starting with 0.5% (vol/vol) methanol. After 3 h of the initial methanol pulse, 1% (vol/vol) methanol was continuously pumped into the reactor for 66 h at an exponential flow rate defined by the equation y = 3.0062e^0.016x^ for µ = 0.016 h^−1^. Protein production at the end of the batch phase, glucose feeding phase, and following methanol feeding phase were analyzed using capillary gel electrophoresis.

### 2.6. Protein Purification

The culture medium was centrifuged at 8000× *g*, 4 °C, for 30 min using a Beckman Avanti J-20XP centrifuge (Beckman Coulter, Brea, CA, USA). The supernatant was then filtered through a 0.45 µm membrane using the Stericup^®^ vacuum filtration system (Merck, Darmstadt, Germany). The desired protein was further purified using a 5 mL HisTrap HP column (GE Healthcare, Chicago, IL, USA), using the ÄKTA^TM^ Protein Purification System (GE Healthcare, Chicago, IL, USA). According to the manufacturer’s instructions, the filtered supernatant was diluted 1:1 with binding buffer (20 mM sodium phosphate pH 7.4 with 0.5 M NaCl and 5 mM imidazole) for sample preparation. For the purification process, the column was equilibrated with five column volumes of binding buffer and a protein sample was loaded into the column at a flow rate of 3 mL/minute. Unbound molecules were washed away with ten column volumes of binding buffer. The target protein was eluted from the column using ten volumes of a linear gradient elution from 0% to 100% of elution buffer (20 mM sodium phosphate pH 7.4 with 0.5 M NaCl and 500 mM imidazole). The imidazole was removed by a dialysis technique. The purified protein was analyzed by SDS-PAGE and Western blot analyses, and the protein concentration was measured by a Bradford assay. The suspected degraded protein bands, ranging from 17 to 18 kDa, presumably caused by certain types of host proteases, were also characterized by LC-MS/MS. As LC-MS/MS analysis of the suspected protein bands, the peptide separations were carried out using a Thermo Dionex Nano LC Ultimate 3000 system equipped with an Acclaim PepMap RSLC C18 column (75 µm × 15 cm, 2 µm particle size, 100 Å pore size, Thermo Scientific, Waltham, MA, US). The 0.1% formic acid in 2% acetonitrile (solvent A) and 0.1% formic acid in 80% acetonitrile (solvent B) were used as mobile phase with the linear gradient condition at the flow rate of 0.3 µL/min. Subsequently, mass spectrometric analysis was performed using a SCIEX Triple TOF 6600+ system (AB SCIEX, Framingham, MA, USA) operating in positive ion mode. Peptide masses were selected at MS 350–1500 Da and the 50 candidate ions signal per cycle which exceeds 100 count per second were subjected to do MS2 at 100–1500 Da. The fragment spectra (MS^2^) were compared against theoretical spectra in a protein database. 

### 2.7. SDS-PAGE and Western Blotting

The protein samples were separated on 13% polyacrylamide gel and transferred onto nitrocellulose membrane, 0.45 µm, using TRANS-BLOT^®^ SD semi-dry transfer cell (BIO-RAD, Hercules, CA, USA). The blot was blocked with blocking solution (1xTBS containing 0.1% Tween-20, 5% skim milk) at 4 °C for 18 h to prevent non-specific binding. Subsequently, the blotting membrane was incubated with anti-6xHis tag Ab linked AP (1:300 in blocking solution) (Invitrogen, Waltham, MA, USA) at room temperature for 2 h prior to washing with TBST for three times. The protein band was visualized using AP conjugate substrate kit (BIO-RAD, Hercules, CA, USA). 

### 2.8. Analysis of Hyaluronidase Activity Assay

The hyaluronidase activity was determined using a turbidity assay conducted in a 1.5 mL microtube. The reaction mixture contained 2 µg of sample protein and 0.5 mg/mL hyaluronic acid in a 0.2 M formate buffer pH 3.0 with 0.15 M NaCl and was incubated at 37 °C for 30 min. The reaction was terminated by adding CTAB reagent (2.5% CTAB in 2.0% NaOH) and further incubating at 37 °C for 10 min. Absorbance was measured at 405 nm using a SPECTROstar Nano (BMG LABTECH, Ortenberg, Germany). The turbidity reducing unit, based on international standards, was calculated as the amount of hyaluronidase required to reduce the turbidity of 50 µg of hyaluronic acid by 50% [21]. The optimal temperature and pH of VesT2a protein were performed at various temperatures ranging from 4 °C to 95 °C and pH systems ranging from pH 2 to pH 10. For the inhibition assay, gallic acid and quercetin were tested as hyaluronidase inhibitors. The inhibitor, at concentrations ranging from 0 to 100 µg/mL, was added to the reaction mixture and incubated at 37 °C for 30 min before stopping the reaction and measuring turbidity at 405 nm.

### 2.9. Molecular Docking

The three-dimensional protein structure prediction of VesT2a was performed using the SWISS-MODEL, a web-based integrated service dedicated to building a homology model of the protein of interest based on the SWISS-MODEL template library as follows: https://swissmodel.expasy.org/interactive (accessed on 31 October 2023). The 3D structure, retrieved from the SWISS-MODEL, was used as the representative model of the hyaluronidase enzyme. Two bioinformatic tools were employed: NetNglyc 1.0 from the DTU Health Tech server (accessed on 11 November 2023 at https://services.healthtech.dtu.dk/services/NetNGlyc-1.0/) for predicting N-glycosylation sites, and Disulfide by Design 2.0 (accessed on 11 November 2023 at http://cptweb.cpt.wayne.edu/DbD2/index.php) for predicting disulfide bond formation. Molecular docking study was carried out using CB-Dock 2 server (accessed on 7 December 2023 at https://cadd.labshare.cn/cb-dock2/php/index.php) based on AutoDock Vina and GOLD Suite 5.2.2 (Genetic Optimization of Ligand Docking), with the software running on an AMD Ryzen 7, 3700U processor with Radeon Vega Mobile Gfx 2.30 GHz, 8.00 GB RAM, and a 64-bit operating system. The putative binding site between VesT2a protein and hyaluronic acid hexamer (PDB ID: 4hya) was predicted using the protein–ligand blind docking tool, CB-Dock 2 server. The selected PDB file of protein–ligand complex was subjected to self-docking to determine optimal parameters for protein–ligand docking, aiming for an RMSD value less than 2 Å, following the GOLD program user manual (accessed on 10 December 2023 at https://www.ccdc.cam.ac.uk/media/Documentation/0C5D99BC-7CC3-49B6-8319-06BEA8CA342D/GOLD_User_Guide_2020_1.pdf). These optimal parameters were then set as the default for docking other ligands, e.g., hyaluronic acid, quercetin, and gallic acid, with VesT2a protein. The protein–ligand complex with the highest fitness score was selected for further evaluation. The BIOVIA Discovery Studio 2021 Client software was utilized to define and visualize the predicted interactions between the VesT2a protein and ligands.

## 3. Results

### 3.1. Construction of Recombinant VesT2a Plasmids and Small-Scaled Expression in P. pastoris

**Figure 2:**
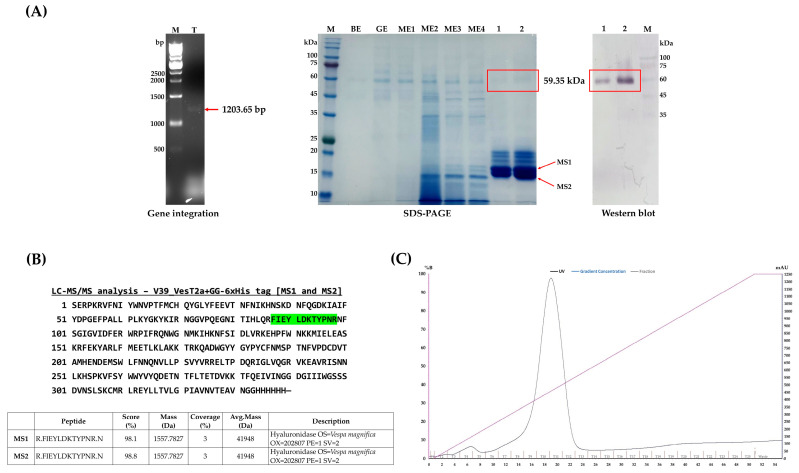
Scale-up production of recombinant VesT2a protein using *P. pastoris*. (**A**) Integration plasmid verification via colony PCR, using 5′AOX1 and 3′AOX1 primers; M: 1kb DNA ladder marker; T: tested colony. The production of VesT2a protein was analyzed using SDS-PAGE and Western blotting techniques; M: protein molecular weight marker; BE: batch end phase; GE: glucose feeding end phase; M1–M4: samples collected during the methanol feeding phase; 1 and 2: VesT2a protein obtained after the purification step at 1× and 2× concentrations (red boxes), respectively. (**B**) Protein sequencing analysis via the LC-MS/MS technique of MS1 and MS2 fragments showed the amino acid sequence highlighted in green. (**C**) The chromatogram of VesT2a protein purification analysis using affinity column chromatography.

We succeeded in converting the mutant type of hyaluronidase (*mVest2a*) into its corresponding wild-type (993 bp; N107D and Q109E) via site-directed mutagenesis. The target fragment (1305 bp) was also included the alpha mating factor and a His-tag (6xHis) at its 5′ and 3′ ends for extracellular secretion and detection/purification, respectively (Figure S3). After successful propagation using the *E. coli* expression (BB1) vector, DNA sequencing confirmed that the coding sequence could be incorporated into the yeast expression (BB3) vector, which was mediated by *Bpi*I, yielding two different plasmids: pGAP_αMF_*VesT2a*_GG-6xHis tag_ScCYC1tt and pAOX1_αMF_*VesT2a*_GG-6xHis tag_ScCYC1tt (Figure S1). Both constructs were designed to compare the efficiency of protein expression in *P. pastoris*. Having been verified to be integrated into the host genome (Figure 2A), small-scale expression in a 96-DWP clearly showed that the highest yield of secreted VesT2a was detected in the clones bearing the P*_AOX1_*; also, the yield was approximately 2.16-fold higher than that of clones bearing the constitutive promoter (Table 3), indicating that a methanol-induced system is needed to achieve a satisfying product titer. Therefore, the clones bearing pAOX1_αMF_*VesT2a*_GG-6xHis tag_ScCYC1tt were chosen for investigating VesT2a production throughout this study.

### 3.2. Upscaled Production, Detection, and Purification of Vest T2a Produced by P. pastoris

*P. pastoris* harboring pAOX1_αMF_*VesT2a*_GG-6xHis tag_ScCYC1tt was then subjected to scaled-up production in a bioreactor system to evaluate its potential as a microbial cell factory for industrial purposes. After 72 h of cultivation under methanol-fed conditions (MeOH; 1% *v/v*), the engineered yeast cells secreted a protein band of approximately 59.35 kDa into the culture medium, with a total protein yield of 96.48 mg/L. The protein was purified using an IMAC column, eluted with 500 mM imidazole, and visually detected using a monoclonal antibody specific to the His-tag fusion protein with an alkaline phosphatase (AP) substrate (Figure 2A). Notably, its size differs from the theoretically calculated value of VesT2a (~40.05 kDa); presumably, it was glycosylated by the yeast cells (Figure 1A). Although we are capable of harnessing *P. pastoris* to produce VesT2a as a soluble and secreted protein, it is suspected that the protein may be subject to undesirable degradation by certain proteases (e.g., Kex2 and Yps1p), as degraded proteins with sizes between 17 and 18 kDa were detected (Figure 2A). Protein sequencing analysis using LC-MS/MS also confirmed that their sequences precisely matched those of hyaluronidase enzymes from *V. tropica* and *V. magnifica* (Figure 2B). Thus, optimizing culture conditions to minimize the unwanted effects of endogenous protein-degrading enzymes is necessary to improve product titer in the long run.

### 3.3. Hyaluronidase Activity Assay and Molecular Docking

**Figure 3:**
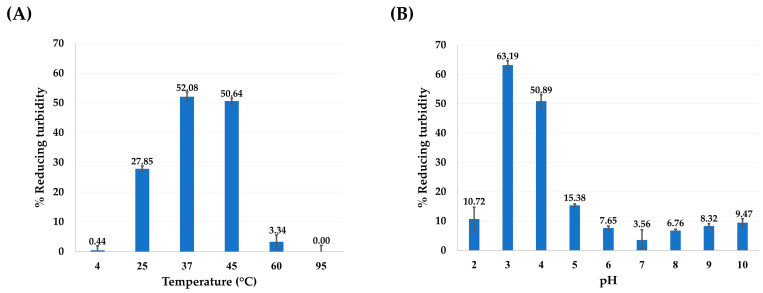
Effects of temperature (**A**) and pH (**B**) on hyaluronidase activity of VesT2a protein.

**Figure 4:**
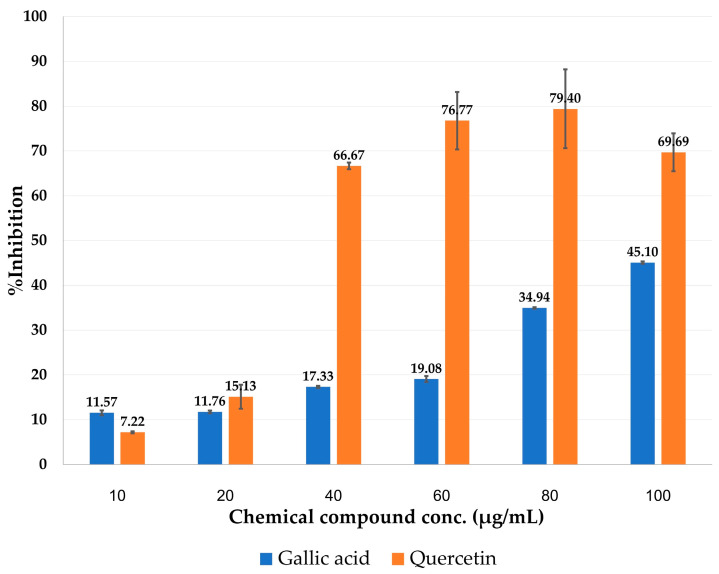
Inhibition of hyaluronidase activity of VesT2a protein by gallic acid and quercetin.

To confirm the functional expression of the recombinant VesT2a, the purified protein fraction containing the target protein band (~59.35 kDa) was tested for hyaluronidase activity using the CTAB turbidimetric method. As anticipated, the active fraction exhibited hyaluronidase activity over a broad range of pH (2–10) and temperatures (4–60 °C). The VesT2a-containing fraction exhibited maximal hyaluronidase activity at pH 3.0, while its activity gradually decreased above this optimal pH value. The enzyme was fully active at 37 °C, but its hyaluronidase activity was absent at 95 °C, indicating protein denaturation due to high temperature sensitivity (Figure 3). To evaluate the unit enzyme activity of the hyaluronidase protein, 0.1 µg of VesT2a protein was tested under optimal conditions, showing a hyaluronidase activity of 4238.37 U/mg. Remarkably, the specific activity was significantly higher than that of the crude venom of *V. tropica* and *E. coli*-derived VesT2a protein, showing 47.49-fold and 148.92-fold differences, respectively (Table 4). Since hyaluronidase is known to be inhibited by various phenolic substances [37], this information was used to characterize the VesT2a produced by the engineered *P. pastoris* strain. Co-incubation with various concentrations of gallic acid and quercetin (0–100 µg/mL) significantly decreased the hyaluronidase activity of the purified protein fraction. Quercetin exhibited a stronger inhibitory effect than gallic acid, implying that the yeast-derived protein is a hyaluronidase enzyme (Figure 4). 

**Figure 5:**
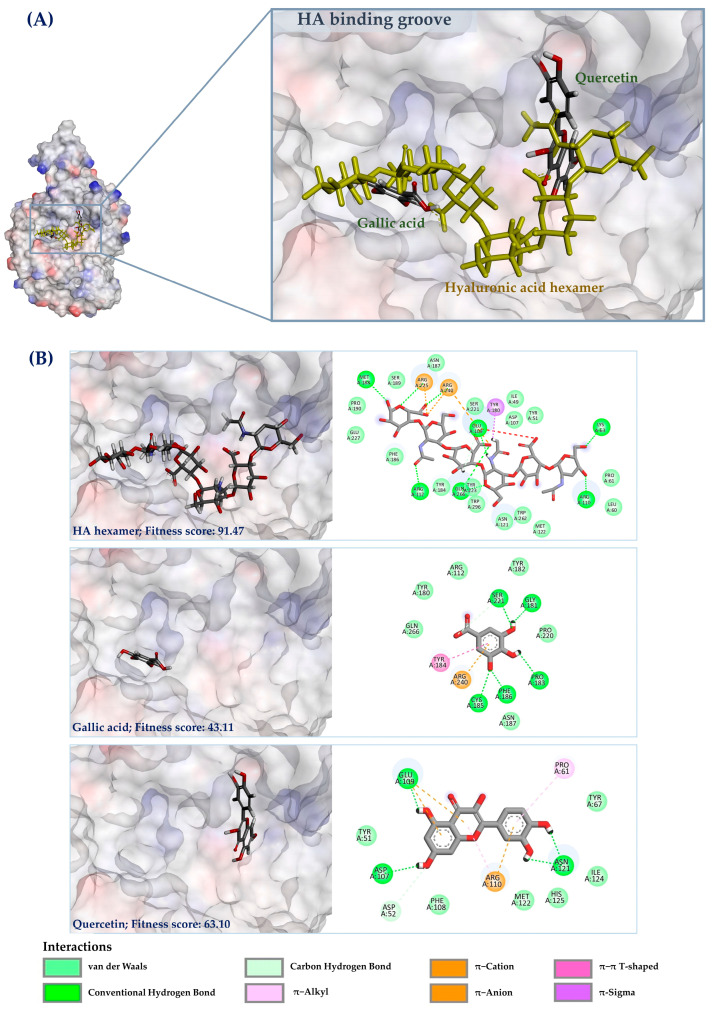
Molecular docking analysis represents the structure orientation of VesT2a protein with a hyaluronic acid hexamer and hyaluronidase inhibitors (**A**) and 2D and 3D ligand interactions diagrams (**B**).

Molecular docking was performed to elucidate the receptor–ligand interaction between the His-tagged hyaluronidase protein (VesT2a) and hyaluronic acid (HA-hexamer). As depicted in Figure 5, the HA-hexamer can interact with the HA binding grooves by forming hydrogen bonds and van der Waals force with various active residues, especially Glu109 (1.82 Å) with Asp107, which are both believed to play crucial roles as proton donors and acceptors in the hyaluronic acid degradation process [38,39]. Meanwhile, the substrate can form the promising π–sigma interaction (2.36 Å) with adjacent amino acids in the active region. In silico dockings also confirmed the inhibitory roles of quercetin and gallic acid as the selective inhibitors of VesT2a. Based on the two hydroxy groups located at the A-ring system, the former, with a binding score of 63.10, formed hydrogen bond interactions with Asp107 and Glu109 (1.82–1.87 Å), while engaging in π–alkyl, π–anion, and π–alkyl interactions with other adjacent residues, including Glu109, Arg110, and Pro61 (3.87–5.22 Å). The latter (fitness score = 43.11), on the other hand, was predicted to interact with the substrate binding region, where it did not interact with either Asp107 or Glu109, suggesting that both phenolic compounds exert different modes of action in the inhibition of the hyaluronidase enzyme.

## 4. Discussion

Hyaluronidases have a wide range of biomedical applications, such as cosmetic surgery, drug delivery dermatology, and aesthetic medicine [10,16,40]. In 2016, our team successfully cloned the gene encoding *VesT2a*; however, heterologous expression of this gene in *E. coli* requires multiple steps to achieve functional re-folding of the protein from inclusion bodies, which has limited its biomedical applications. Additionally, the recombinant protein exhibited low catalytic activity, even when fused with soluble tags (e.g., Thioredoxin/Trx•Tag™) and expressed at lower temperatures ranging from 15–20 °C. Given that this protein contains putative glycosylation sites (Asn79, Asn99, Asn127, Asn187, and Asn325) and several cysteines in its sequence (Figure 1F), we hypothesized that further modifications, such as disulfide bond formation and glycosylation, are necessary for proper folding and solubility. Among microbial expression systems, *P. pastoris* offers numerous advantages, including the ability to facilitate disulfide bond formation and glycosylation, both of which are vital for the successful production of eukaryote-derived proteins [23,41,42]. Moreover, it can be practically engineered to produce secreted proteins using leader sequences such as the human serum albumin (HSA) signal, mating factor α1 (α-MF), and killer toxin signal sequence [43,44]. This allows the target proteins to be harvested directly from the culture medium, eliminating the need for labor-intensive and costly downstream processes like cell disruption step. For these reasons, *P. pastoris* was chosen as the microbial chassis to foresee whether it can produce insect-derived hyaluronidase in our study. Although *V. tropica* and *P. pastoris* are both classified as eukaryotic species, they differ moderately in codon usage, approximately 22.66%, with a codon adaptation index (CAI) value of 0.75. This suggests that codon optimization is still necessary to ensure proper expression in the yeast system (Figure S2). Our assumption is likely supported by Reitinger [32] and colleagues, who demonstrated that the use of a codon-optimized version of the bee-derived hyaluronidase resulted in higher catalytic activity compared to the wild-type gene. The codon-optimized VesT2a, achieved through gene synthesis technology, was hence implemented in this study. 

Golden*PiCS* is a flexible and efficient gene construction method specifically designed for heterologous protein expression and pathway engineering in *P. pastoris* [35]. Throughout this strategy, multiple pieces of target DNAs, e.g., gene of interest, promoters, and terminators, could be assembled within a few steps, meaning it reduces time consumption, minimizes chemical consumption, and is much more cost-effective. Based on the unique and specialized cutting efficacy of two restriction enzymes—*Bsa*I and *Bpi*I—the *VestT2a* gene was successfully incorporated into the *P. pastoris* expression vector (designated as dBB3), resulting in the construction of pGAP-αMF-*VesT2a* and pAOX1-αMF-*VesT2a* (Figure S1) in a short period of time. Having been integrated into the yeast genome through homologous recombination (Figure 2A), the glucose release assay showed that clones bearing the recombinant plasmid pAOX1-αMF-*VesT2a* produced approximately 2.16 times more secreted VesT2a than those carrying the constitutive promoter. This result clearly indicates that strong induction is required to effectively drive the high expression of VesT2a in *P. pastoris* controlled under *AOX* promoter. As per numerous findings, the distinct expression levels between induced and non-induced promoters have been well-documented where the tightly regulated *AOX* promoter, driven by methanol, frequently achieves significantly higher expression levels compared to the constitutive *GAP* promoter [45,46,47]. Another reason for this might stem from the fact that the methanol induction system enables post-growth activation in the *Pichia* system, redirecting metabolic and cellular resources (e.g., amino acids and cofactors) away from biomass formation toward heterologous protein synthesis, thereby enhancing yield during the induction phase [48,49]. Although no relevant information supports the obtained results yet, a significant difference in expression levels between the *GAP* and *AOX* promoters was observed, with the inducible promoter leading to high-level expression of β-fructofuranosidase in *P. pastoris* [50].

The clone bearing pAOX1-αMF-*VesT2a* was then subjected to upscaled production to see whether it could shed some light on industrial applications. After being fed with methanol (1% *v/v*) for 72 h, the engineered yeast produced a secreted protein, suspected to be a glycosylated form of VesT2a, as detected by Western blot analysis (59.35 kDa). This glycosylation likely stems from the enzyme bears putative glycosylated asparagine residues at positions 79, 99, 127, 187, and 325 (Figure 1F). Our results are clearly consistent with previous findings [33,51], which demonstrated that the scorpion venom hyaluronidase (rTsHal-1), containing five putative N-glycosylation sites, as well as human granulocyte-macrophage colony-stimulating factor (hGM-CSF), were expressed as glycosylated forms, showing a 16 kDa increase in size when heterologously expressed in *P. pastoris*. In fact, hyperglycosylation of recombinant proteins produced by *P. pastoris* serves as a common issue, where they typically acquire additional glycan sites compared to their native counterparts. This involves the addition of high-mannose-type N-glycans, leading to increased size, stability, and changes in the function of the proteins [52,53]. However, to confirm the probable glycosylation issue of VesT2a produced by the methylotrophic yeast, it will be necessary, in upcoming experiments, to employ techniques such as glycosidase enzymes to cleave the recombinant protein and verify the existence of a size-reduced form, thereby consolidating the current results. Despite successful production in *P. pastoris*, VesT2a was suspected to undergo unexpected degradation by endogenous proteases such as aspartyl protease (Yps1p) and Kex2 protease. These proteases are primarily active at the plasma membrane and, under some conditions, may be secreted into the extracellular medium, where they recognize paired basic amino acid residues (KR or RR) as potential cleavage sites. This degradation not only reduces the yield and activity of secretory proteins but also complicates and laborites the separation and purification processes, thereby hindering long-term industrial applications [54]. Previous studies, however, show that constructing protease-deficient strains or supplying external protease inhibitors can alleviate this problem and lead to significantly improved product titers. For instance, Liu et al. [54] knocked out multiple endogenous proteases in *P. pastoris*, which significantly reduced protein degradation and subsequently improved protein titers. Meanwhile, the use of Yps1p-deficient strains resolved antibody production issues in the methylotrophic yeast *Ogataea minuta* [55]. Accordingly, these strategies will be implemented to ensure a sufficient amount of non-degraded VesT2a (approximately 59.35 kDa) is available for future investigations, such as enzyme kinetic studies, to obtain the most reliable Km and Vmax values, thereby supporting various potential medical uses and structural biology explorations. 

To the best of our knowledge, recombinant VesT2a exhibits the highest hyaluronidase activity among insect-derived proteins produced in yeast expression systems to date [31,32]. Notably, recombinant VesT2a remains active across a broad range of pH and temperature, indicating its potential for diverse applications. According to Lenormand et al. [56], hyaluronidase exhibits an acidic pH optimum near 4 and becomes inactive at pH levels above 5.5 under low salt conditions and in the absence of BSA or LYS. However, the enzyme remains active at pH 7 and up to pH 9 in the presence of high-pI proteins [56]. Similarly, our study suggests this behavior may result from the presence of five basic amino acids, including arginine (positions 110, 112, 225, and 240) and lysine (position 63), within the active site of VesT2a (Figure 5B), which play a critical role in binding negatively charged hyaluronic acid. Under basic conditions, these amino acids may act as proton donors, enhancing electrostatic interactions within the hyaluronidase-HA complex and improving hydrolytic activity. Additionally, its specific activity is significantly higher than that of the original crude venom and the *E. coli*-expressed VesT2a, showing increases of 47.49-fold and 148.92-fold, respectively (Table 4). Even though *E. coli* systems are capable of expressing VesT2a at high levels, the recombinant protein was predominantly detected as an inclusion body, with a size smaller than the expected value and exhibiting low specific activity (Figure S4). This suggests that post-translational modifications, such as disulfide bond formation and proper glycosylation, are essential for the correct folding and solubility of the recombinant protein [31]. The limitations of *E. coli* systems in expressing eukaryotic-derived hyaluronidases (e.g., human and bee) have also been documented, requiring post-translational modifications (e.g., disulfide bond formation and glycosylation) to obtain more active proteins, some of which can be resolved using *P. pastoris* [32,57,58]. While it is clear that Hyals, including wild-type VesT2a, belongs to hyaluronate 4-glucanohydrolase (EC 3.2.1.35) and naturally generates tetra- and hexamer-HA as end products from high-molecular-weight HA [1], the VesT2a produced by *P. pastoris* may display different catalytic activities; this is particularly the case regarding the types of end products occurred, due to its GG-6His tag and distinct glycosylation pattern. Therefore, identifying the resulting HA oligomers is crucial as we move forward to ensure the most precise application.

According to the in silico analyses, the potential structure of VesT2a (as a His-tag fusion protein), generated by SWISS-MODEL, can interact with the HA hexamer specifically in binding grooves by forming hydrogen bonds and van der Waal force with several key residues, particularly Glu109 (1.82 Å) and Glu109. These residues, with pKa values of 3.90 and 4.07, respectively, play crucial roles as proton donors and acceptors [38,39], facilitating optimal substrate degradation under acidic conditions (pH 3). A previous study conducted by Lee and Kim [59] demonstrated that flavonoids act as promising inhibitors of hyaluronidase enzymes by strongly interacting with the active residues in the HA binding groove. Our in vitro and in silico analyses also clearly demonstrated that quercetin, a key flavonoid commonly found in many plant species, effectively inhibits VesT2a activity and is predicted to bind at the enzyme’s catalytic site, highlighting its future potential of plant-based extracts for alleviating symptoms in patients suffering from insect bites and stings.

## 5. Conclusions

Implementing *P. pastoris* as the microbial expression system, we demonstrated that the gene encoding VesT2a could be functionally produced as an extracellularly secreted protein, thereby resolving the inclusion body formation typically observed in *E. coli* expression systems. The recombinant VesT2a, presented in its glycosylated form, shows higher hyaluronidase activity compared to its original crude venom and the corresponding recombinant protein expressed in the *E. coli* system. This suggests that post-translational modifications, such as disulfide bond formation and glycosylation, are essential for maintaining the protein’s function. The use of molecular docking has revealed its potential molecular mode of action, where the HA hexamer interacts effectively with both the catalytic and substrate recognition sites of the recombinant enzyme, providing insights for further protein engineering to enhance its catalytic activity. Moreover, gallic acid and quercetin demonstrate hyaluronidase inhibitory effects, confirming that the secreted protein is indeed a hyaluronidase. Owing to its promising catalytic activity across a diverse range of physiological conditions, the successful expression of VesT2a through the yeast expression system not only paves the way for various medical applications but also illuminates the potential for determining its specific inhibitors from natural resources.

## Figures and Tables

**Table 1:** Specific primers for hyaluronidase gene construction.

Name	Sequence (5′ to 3′)	Purpose
F_VesT2a	GATCGGTCTCGTCAGAAAGGCCCAAAAGAGTGTTTAACATTTACTG	VesT2a gene
R_VesT2a	GATCGGTCTCCAAGCCTATTAGTGATGGTGGTGGTGATGTCCAC	VesT2a gene
F_SDM	GGCGTGATAGACTTCGAAAGATGGC	Gene mutation **
R_SDM	ACGCCATCTTTCGAAGTCTATCACG	Gene mutation **
F_BB1	CAGGAAACAGCTATGAC	Sequencing
R_BB1	GTAAAACGACGGCCAGTT	Sequencing
F_AOX1	CTTTCATAATTGCGACTGGTTC	Sequencing
F_GAP	ACCAGAATCGAATATAAA	Sequencing
R_BB3	CGAGCGTCCCAAAACC	Sequencing
5′AOX1	GACTGGTTCCAATTGACAAGC	Gene integration
3′AOX1	GCAAATGGCATTCTGACATCC	Gene integration

** The underlines are the mutated codon for an amino acid.

**Table 2:** Plasmid DNA for Golden Gate derived *P. pastoris* cloning system (Golden*PiCS*).

Plasmid DNA Name	Purpose
eBB1_Fs-23	Empty vector of backbone 1 (eBB1)
BB1_12_pGAP	Constitutive promoter
BB1_12_pAOX1	Inducible promoter
BB1_34_ScCYC1tt	Terminator
edBB3aZ_Fs-14	Empty vector of direct backbone 3 (edBB3)

**Table 3:** Screening of recombinant VesT2a protein productions using enzymatic glucose release technique.

Construction	Promoter	Terminator	Protein Conc. (mg/L)
VesT2a + GG-6xHis tag	P_GAP_	ScCYC1tt	8.91
VesT2a + GG-6xHis tag	P*_AOX1_*	ScCYC1tt	19.23 (2.16-folds)

**Table 4:** Comparative of specific hyaluronidase activity.

	Crude Venom	VesT2a (*E. coli*)	VesT2a (*P. pastoris*)
**Specific activity** (U/mg)	89.25 ± 4.15	28.46 ± 0.71	4238.37 ± 135.65

## Data Availability

All data supporting the conclusions of this article are included in this article.

## Supplementary Materials

The following supporting information can be downloaded at: https://www.mdpi.com/article/10.3390/jof10120854/s1, Figure S1: The recombinant plasmids of *VesT2a* gene; BB3aZ_Fs-14_pGAP_αMF_*VesT2a*_GG-6xHis tag_ScCYC1tt and BB3aZ_Fs-14_pAOX1_αMF_*VesT2a*_GG-6xHis tag_ ScCYC1tt; Figure S2: Comparison of DNA sequences of hyaluronidase (*VesT2a*) gene between the original DNA sequence derived from *V. tropica* venom and codon optimized *VesT2a*; Figure S3: The synthetic gene of the coding sequence (CDS); Figure S4: SDS-PAGE and Western blot analysis of hyaluronidase derived from *V. trpoica* venom (VesT2a) in different expressions.
