# Supplementary material for: Production of Vespa tropica Hyaluronidase by Pichia pastoris
Source: J Fungi (Basel). 2024 Dec 11;10(12):854. doi: 10.3390/jof10120854 (PMC11676926; doi:10.3390/jof10120854)
Supplement: Supplementary file 1 [file jof-10-00854-s001.zip › jof-3317009-supplementary.pdf]

Supplementary materials

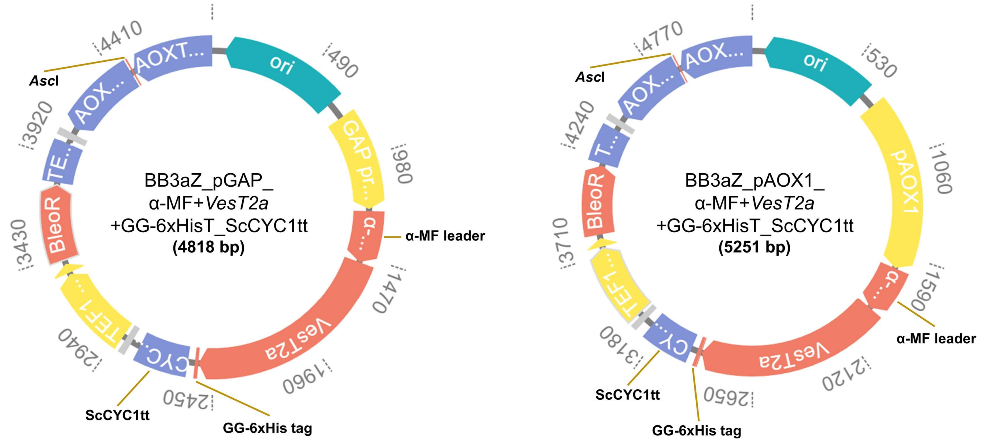

Figure S1. The recombinant plasmids of *VesT2a* gene; BB3aZ_Fs-14_pGAP_αMF_*VesT2a*_GG-6xHis tag_ScCYC1tt and BB3aZ_Fs-14_pAOX1_αMF_*VesT2a*_GG-6xHis tag_ ScCYC1tt

(*V. tropica*)	001	tcc gag aga ccg aaa aga atc ttc aac att tat tgg aat gtt cct acc ttt atg tgt cat	060
(Co-optimized)	001	tca gaa agg ccc aaa aga gtg ttt aac att tac tgg aac gta ccc act ttc atg tgt cac	060
(*V. tropica*)	061	caa tat ggt ctc tac ttc gag gag gtg aca aat ttt aat ata aaa cat aat tct aag gac	120
(Co-optimized)	061	caa tat ggt ctt tat ttt gag gag gtt act aac ttc aat att aag cat aat tca aag gat	120
(*V. tropica*)	121	gat ttc cag gga gac aag ata gca att ttt tat gat cct gga gaa ttc ccg gcg tta ttg	180
(Co-optimized)	121	aac ttc caa ggc gat aaa atc gca atc ttc tat gat cct ggt gag ttt ccc gct ttg ctg	180
(*V. tropica*)	181	cca ctc aaa tat ggc aaa tat aag ata aga aac ggt gga gtt cct caa gaa ggt aac ata	240
(Co-optimized)	181	cct ttg aaa tac gga aaa tac aag atc cgt aac ggt ggt gta cca caa gaa gga aac att	240
(*V. tropica*)	241	acg ata cat ctt caa aga ttt atc gaa tat ttg gat aaa aca tat cca aat aga aac ttc	300
(Co-optimized)	241	aca atc cat tta caa aga ttt atc gag tac ctt gac aaa aca tat cct aat aga aac ttc	300
(*V. tropica*)	301	agt ggt atc ggt gta atc gac ttt gaa aga tgg aga cct atc ttc cga caa aat tgg ggc	360
(Co-optimized)	301	tca ggt atc ggc gtg ata gac ttc gaa aga tgg cgt cca ata ttt agg caa aat tgg ggt	360
(*V. tropica*)	361	aat atg aag ata cat aag aat ttt tca ata gat tta gtt cgc aaa gaa cat cca ttc tgg	420
(Co-optimized)	361	aat atg aaa ata cat aaa aat ttc tcc ata gac cta gtt cgt aag gaa cat cca ttc tgg	420
(*V. tropica*)	421	aat aaa aag atg atc gaa ttg gag gca tcg aag agg ttc gaa aaa tat gcc aga ctt ttc	480
(Co-optimized)	421	aac aaa aaa atg atc gag ctt gag gcc tca aag aga ttt gaa aag tac gca cgt cta ttc	480
(*V. tropica*)	481	atg gag gaa act ttg aaa ttg gcc aaa aag act agg aag caa gct gat tgg ggt tat tat	540
(Co-optimized)	481	atg gag gaa acg ctt aaa ctt gcc aaa aaa acg agg aag caa gct gac tgg ggt tac tac	540
(*V. tropica*)	541	gga tat ccc tat tgc ttt aat atg tcg cct act aat ttc gtc cct gat tgt gat gtt aca	600
(Co-optimized)	541	ggt tac cca tat tgc ttt aat atg tca cca acc aat ttt gta cct gac tgt gac gta aca	600
(*V. tropica*)	601	gcg atg cac gag aac gac gag atg tcg tgg ctg ttc aat aat caa aat gta ctt cta cca	660
(Co-optimized)	601	gcc atg cac gaa aat gat gaa atg agt tgg tta ttc aac aat cag aac gtc tta tta cca	660
(*V. tropica*)	661	tcc gtc tac gtt aga cgt gaa ctg acc cct gat caa aga att ggt tta gtg caa gga aga	720
(Co-optimized)	661	tct gtc tat gtg cgt aga gag ttg acg cct gac cag aga ata gga ttg gtt cag ggc aga	720
(*V. tropica*)	721	gta aag gaa gct gtt agg atc tcg aac aat tta aag cat tca ccg aaa gtg ttc tct tat	780
(Co-optimized)	721	gtt aaa gaa gcc gta agg atc tcc aac aac tta aag cac agt cct aag gtc ttt agt tat	780
(*V. tropica*)	781	tgg tgg tac gtg tat cag gac gaa acg aat act ttt ctt acc gag acc gac gtg aaa aag	840
(Co-optimized)	781	tgg tgg tat gtg tac caa gac gaa act aac acg ttt tta acg gaa act gat gtc aaa aaa	840
(*V. tropica*)	841	act ttc caa gag ata gtg ata aac ggt ggg gat ggt atc att ata tgg ggt agc tcg tcc	900
(Co-optimized)	841	acc ttc cag gaa att gtg att aac ggt gga gac ggc ata att ata tgg ggc tca tct tca	900
(*V. tropica*)	901	gac gta aac agc tta agt aaa tgt atg aga ttg cgg gag tat ctg tta acg gtt tta gga	960
(Co-optimized)	901	gat gtc aac agt ctg agt aaa tgc atg cgt ctg aga gag tac tta ttg act gtg ctt ggc	960
(*V. tropica*)	961	cca atc gcg gtt aac gtg aca gaa gcc gtt aac	
(Co-optimized)	961	ccc atc gct gtg aat gtt acc gaa gct gtt aac	

Figure S2. Comparison of DNA sequences of hyaluronidase (*VesT2a*) gene between the original DNA sequence derived from *V. tropica* venom (black) and codon optimized *VesT2a* gene (red).

```
     α-MF reader
1    atg cgt ttt cca agt att ttc aca gcc gta cta ttc gcc gca tcc agt gct cta gca gcc cca gtg aat act acc   75
1     M   R   F   P   S   I   F   T   A   V   L   F   A   A   S   S   A   L   A   A   P   V   N   T   T    25
76   acc gag gac gag aca gcc cag att cct gcc gaa gca gtg atc ggt tac agt gat cta gaa gga gat ttc gac gtc   150
26    T   E   D   E   T   A   Q   I   P   A   E   A   V   I   G   Y   S   D   L   E   G   D   F   D   V    50
151  gct gtc ctg ccc ttc tca aat tct acg aac aac ggc ctg ctg ttt att aat act act ata gct tcc atc gcc gcc   225
51    A   V   L   P   F   S   N   S   T   N   N   G   L   L   F   I   N   T   T   I   A   S   I   A   A    75
                                           Kex2p cleavage site
                                                               VesT2a
226  aag gaa gag ggc gta tct cta gaa aag cgt tca gaa agg ccc aaa aga gtg ttt aac att tac tgg aac gta ccc   300
76    K   E   E   G   V   S   L   E   K   R   S   E   R   P   K   R   V   F   N   I   Y   W   N   V   P    100
301  act ttc atg tgt cac caa tat ggt ctt tat ttt gag gag gtt act aac ttc aat att aag cat aat tca aag gat   375
101   T   F   M   C   H   Q   Y   G   L   Y   F   E   E   V   T   N   F   N   I   K   H   N   S   K   D    125
376  aac ttc caa ggc gat aaa atc gca atc ttc tat gat cct ggt gag ttt ccc gct ttg ctg cct ttg aaa tac gga   450
126   N   F   Q   G   D   K   I   A   I   F   Y   D   P   G   E   F   P   A   L   L   P   L   K   Y   G    150
451  aaa tac aag atc cgt aac ggt ggt gta cca caa gaa gga aac att aca atc cat tta caa aga ttt atc gag tac   525
151   K   Y   K   I   R   N   G   G   V   P   Q   E   G   N   I   T   I   H   L   Q   R   F   I   E   Y    175
                                                                ***     ***
526  ctt gac aaa aca tat cct aat aga aac ttc tca ggt atc ggc gtg ata gac ttc gaa aga tgg cgt cca ata ttt   600
176   L   D   K   T   Y   P   N   R   N   F   S   G   I   G   V   I   D   F   E   R   W   R   P   I   F    200
601  agg caa aat tgg ggt aat atg aaa ata cat aaa aat ttc tcc ata gac cta gtt cgt aag gaa cat cca ttc tgg   675
201   R   Q   N   W   G   N   M   K   I   H   K   N   F   S   I   D   L   V   R   K   E   H   P   F   W    225
676  aac aaa aaa atg atc gag ctt gag gcc tca aag aga ttt gaa aag tac gca cgt cta ttc atg gag gaa acg ctt   750
226   N   K   K   M   I   E   L   E   A   S   K   R   F   E   K   Y   A   R   L   F   M   E   E   T   L    250
751  aaa ctt gcc aaa aaa acg agg aag caa gct gac tgg ggt tac tac ggt tac cca tat tgc ttt aat atg tca cca   825
251   K   L   A   K   K   T   R   K   Q   A   D   W   G   Y   Y   G   Y   P   Y   C   F   N   M   S   P    275
826  acc aat ttt gta cct gac tgt gac gta aca gcc atg cac gaa aat gat gaa atg agt tgg tta ttc aac aat cag   900
276   T   N   F   V   P   D   C   D   V   T   A   M   H   E   N   D   E   M   S   W   L   F   N   N   Q    300
901  aac gtc tta tta cca tct gtc tat gtg cgt aga gag ttg acg cct gac cag aga ata gga ttg gtt cag ggc aga   975
301   N   V   L   L   P   S   V   Y   V   R   R   E   L   T   P   D   Q   R   I   G   L   V   Q   G   R    325
976  gtt aaa gaa gcc gta agg atc tcc aac aac tta aag cac agt cct aag gtc ttt agt tat tgg tgg tat gtg tac   1050
326   V   K   E   A   V   R   I   S   N   N   L   K   H   S   P   K   V   F   S   Y   W   W   Y   V   Y    350
1051 caa gac gaa act aac acg ttt tta acg gaa act gat gtc aaa aaa acc ttc cag gaa att gtg att aac ggt gga   1125
351   Q   D   E   T   N   T   F   L   T   E   T   D   V   K   K   T   F   Q   E   I   V   I   N   G   G    375
1126 gac ggc ata att ata tgg ggc tca tct tca gat gtc aac agt ctg agt aaa tgc atg cgt ctg aga gag tac tta   1200
376   D   G   I   I   I   W   G   S   S   S   D   V   N   S   L   S   K   C   M   R   L   R   E   Y   L    400
                                                                                 GG-6xHis tag
1201 ttg act gtg ctt ggc ccc atc gct gtg aat gtt acc gaa gct gtt aac ggt gga cat cac cac cac cat cac taa   1275
401   L   T   V   L   G   P   I   A   V   N   V   T   E   A   V   N   G   G   H   H   H   H   H   H   -    425
1276 tag                                                                                                   1278
426
```

Figure S3. The synthetic gene of the coding sequence (CDS) is composed of alpha mating factor, hyaluronidase (*VesT2a*) and fusion tag (GG-6xHis tag). The catalytic residues, D107 and E109, were labeled with asterisks (***).

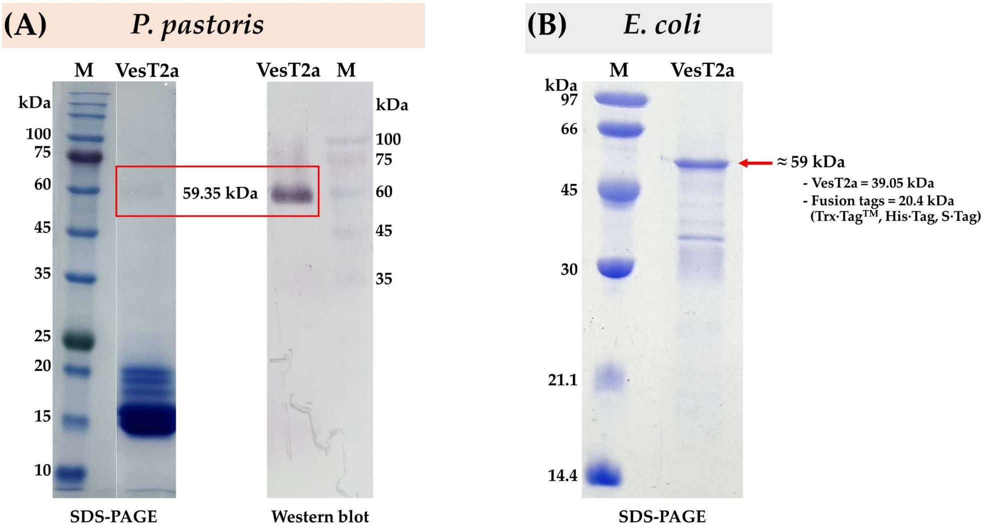

Figure S4. SDS-PAGE and western blot analysis of hyaluronidase derived from *V. trpoica* venom (VesT2a) in different expressions. (A) The MW of VesT2a protein is about 59.35 kDa when expressed in E. coli system; (B) The MW of VesT2a protein with fusion tag (Trx·Tag™, His·Tag, S·Tag) is approximately 59 kDa when expressed in P. pastoris system.
